# Swedish Sport Policy in an Era of Neoliberalism: An Expression of Social Entrepreneurship?

**DOI:** 10.3389/fspor.2021.715310

**Published:** 2021-09-13

**Authors:** Daniel Bjärsholm, Johan R. Norberg

**Affiliations:** ^1^Department of Sport Science, Linnaeus University, Växjö, Sweden; ^2^Department of Sport Sciences, Malmö University, Malmö, Sweden

**Keywords:** austerity, policy development, social entrepreneurial sport policy, social innovation, social innovation policy, sport policy, Sweden

## Abstract

Since the turn of the millennium, Sweden has, like many other countries, become more neoliberal in many areas, including that of sport. The government has increased its expectations on the sports movement and become more result-oriented, which, for example, its revised motives for supporting the sports movement and the establishment of an audit organization can illustrate. However, in contrast to other countries, the Swedish government has not introduced any financial cutbacks in its support for sports. Rather, the opposite is true. The financial support has increased significantly over the last two decades. In the paper, we argue that this contradictory development of Swedish sport policy can be understood as expressions of neoliberalism and social entrepreneurship. As a theoretical concept, social entrepreneurship offers a way of understanding the increased Swedish government support for sport. There are in particular two underlying reasons for this claim. Firstly, sport is considered as a solution to various societal problems, such as social exclusion and refugee crises. Secondly, much of the increased support has been in form of various large-scale, earmarked, and time-limited political initiatives/reforms and project grants, which all have aimed to achieve social change through sport, such as social inclusion. In the paper, we consider these initiatives as expressions of social entrepreneurship. This paper contributes to the ongoing scholarly debate on how neoliberalism and neoliberal policies in the public sector have affected sport organizations. Also, by using social entrepreneurship theory, we provide new theoretical insights into how sport policy can be understood and analyzed.

## Introduction

One of the major political changes that has taken place since the 1970s is the global advancement of neoliberalism and its far-reaching effects on many countries' public welfare systems (Larsson, 2014). The rise of neoliberal ideals in the 1970s is explained, among other things, by recurring global economic crises that led to high rates of unemployment, weak economic growth, and high inflation. During the 1980s, many European countries re-evaluated and transformed their welfare systems: from applying a Keynesian model of pursuing an active and expansive economic stabilization policy in recession to instead introducing austerity policies and cuts in the public sector. In accordance with the economic paradigm of neoliberalism, decentralization, privatization, deregulation, and austerity policies were implemented in many welfare areas (Hermann, 2007; Blyth, 2013; Larsson, 2014). The effects of this neoliberal development can still be seen today (Surender, 2004). A study by Peters (2012) shows, for example, that between 1995 and 2005 (i.e., before both the financial crisis in 2008 and the COVID-19 pandemic in 2020), several of the countries within the international economic organization OECD reduced their public spending by about eight percent of the countries' GDP.

As a result of the neoliberal ideals, the governance and management of the public sector changed. In many parts of the public administration, administrative reforms were carried out, clearly inspired by the corporate sector. Today, these new governance strategies are gathered under the heading of New Public Management (NPM) (e.g., Hood, 1991, 1995; Boston et al., 1996; Eklund and Henrekson, 2011). At the same time, new views on accountability emerged. Previously, political decision-makers had controlled public authorities and services through bureaucratic procedures and rules. Now, public authorities are controlled by clearly defined aims, quantified objectives, and audits (see e.g., Hood, 1991, 1995; Dunleavy and Hood, 1994; Boston et al., 1996; van Kersbergen and van Waarden, 2004; Premfors et al., 2009). The main reason behind this change was that politicians should not govern public operations in detail but rather specify the goals and results that authorities should achieve. As a result, many public authorities, at all political levels, chose to outsource parts of their operations (Kumlin, 2003).

The described neoliberal and economic development have not gone unnoticed by researchers in the fields of sport policy and sport management. Many studies show that sport organizations have been affected by neoliberal austerity policies and their following economic cutbacks (e.g., Giannoulakis et al., 2017; Parnell et al., 2017a, 2018; Roberts, 2017; Walker and Hayton, 2017; Brown and Pappous, 2018; Iversen, 2018). The main impression of the findings is negative. In the studied countries so far in Europe, public support for sport has in general decreased and affected sport organizations and their managerial conditions (e.g., Parnell et al., 2017b; Roberts, 2017; Walker and Hayton, 2017; Iversen, 2018; Widdop et al., 2018), the only exception being the Netherlands which has almost been able to maintain its public support for sport (Hoekman et al., 2018). In some countries, the reduction has been voluntary, for example the UK. In other countries, such as Greece, savings have been enforced as a result of the countries' worrying economic situations (e.g., Parnell et al., 2017a).

Swedish sport policy can, however, be characterized as a deviant case (see Lijphart, 1971; Seawright and Gerring, 2008). Despite recurring economic recessions and neoliberal welfare reforms, the public support for sport has increased significantly, among other things through the implementation of new grants. The actual numbers are striking. At the end of the 1990s, the government support for sports was ~SEK 450 million. Today, the annual support amounts to more than SEK 2 billion (Norberg, 2019). Although public support for sport has increased, the era of neoliberalism has made clear marks on Swedish sport policy. For example, both the structure of state support and the relationship between the state and the Swedish sports movement have changed (e.g., Bergsgard and Norberg, 2010; Fahlén and Stenling, 2016; Norberg, 2016). These marks are the topic of this paper.

The aims of this paper are two-fold. Firstly, we will show in what ways the era of neoliberalism has influenced Swedish sport policy. In doing so, we will contribute to, and nuance, the ongoing academic discussion on how neoliberal policies in the public sector have affected sport organizations (see e.g., Giannoulakis et al., 2017; Parnell et al., 2017a; Roberts, 2017; Walker and Hayton, 2017; Widdop et al., 2018). Our argument is that the public support for sport in Sweden has increased, but also led to a shift in Swedish sport policy and at the cost of the autonomy of the sports movement. Secondly, we will discuss how Swedish sport policy can be interpreted as a manifestation of social entrepreneurship. By linking social entrepreneurship theory to sport policy research, we aim to, as Ratten (2017) requests, provide new theoretical insights into how to analyze and understand policy-making processes and their implementation in the field of sport (see also Ratten, 2019). More precisely, we argue that the new grants to the sports movement can be interpreted as acts of social entrepreneurship.

In this paper, we synthesize and re-analyze the existing depiction of Swedish sport policy and present it in a new way. Both primary and secondary data are included. The primary data consists of documents published by the Swedish government, such as Budget Bills (Prop.) and Swedish Government Official Reports (SOU series). The secondary data consists of the many studies that have been published on Swedish sport policy and whose data also is based on the just mentioned document types. The two sources are used in the paper to portray the Swedish sport policy and its development, and support the new theoretical insights. In the paper, the previous research and the documents are re-analyzed based on the concepts of neoliberalism, NPM, and social entrepreneurship. Secondary data analysis, such as this, has become more prevalent (Smith, 2008), and a viable option for researchers to offer “interpretations, conclusions or knowledge additional to, or different from, those presented in the first report on the enquiry as a whole and its main results” (Hakim, 1982, p. 12). Furthermore, by re-analyzing previous research from a different theoretical perspective, we show that there always exist “multiple possible true descriptions of a given action or phenomenon” (Shapiro, 2002, p. 604), in this case the Swedish sport policy and its development.

In the following section we will present the concept of social entrepreneurship and relate this concept to policymaking. Thereafter, we provide an historic in-depth presentation of Swedish sport policy and its recent changes, especially since the turn of the millennium. Finally, we discuss what the implications can be of sport policy becoming more influenced by social entrepreneurship.

## Social Entrepreneurship

The concept of social entrepreneurship was uncommon in research until the end of 1990s (Huybrechts and Nicholls, 2012), but has since then gained an increased attention (e.g., Short et al., 2009), including in the field of sport (e.g., Ratten, 2011; Bjärsholm, 2017, 2019; Peterson and Schenker, 2018b; McSweeney, 2020). Much interest has been devoted to defining the concept, yet, there is still no uniform definition of social entrepreneurship (e.g., Defourny and Nyssens, 2010; Bacq and Janssen, 2011; Huybrechts and Nicholls, 2012). However, a majority of researchers agree that a social aim or mission is central in any definition of social entrepreneurship (Huybrechts and Nicholls, 2012; see also Austin et al., 2006; Defourny and Nyssens, 2010); that is, a focus on social outcomes rather than on making financial profits. Also, the approach to achieving the social mission is characterized by innovation in its broad sense (Nicholls, 2006). Additionally, many researchers agree on that all profit should be reinvested in the social entrepreneurial organization instead of being distributed among its stakeholders (Bacq and Janssen, 2011).

Underlying reasons for this increase in research can be found in that social entrepreneurial organizations have been established due to societal challenges (e.g., migration, poverty, inequality, and climate change), or from the failure of institutions, such as the market or the government (Huybrechts and Nicholls, 2012; Santos, 2012; Stanković, 2020). The market is often “considered to be ill-equipped to address social problems” (Yunus, 2006, p. 41) since it does not “do a good job of valuing social improvements, public goods and harms, and benefits for people who cannot afford to pay” (Dees, 1998, p. 3). Governments play an important part in correcting market failures (Santos, 2012). However, as many countries have transformed due to neoliberal thinking (Roper and Cheney, 2005) and NPM-inspired reforms (Hammerschmid et al., 2019), their governments have scarce resources to deal with all externalities which have led to “fewer and different interventions by the public” (Hoogendoorn, 2011, p. 5). As a result, states cannot provide public services or solutions through their government agencies to a sufficient extent. Consequently, there may be social problems or other areas that are either hard, or even impossible, to solve or neglected by the government; that is, a so-called government failure has arisen (Santos, 2012). One should, though, remember that many of the societal challenges are too complex to solve for an individual actor, such as a country, organization, or individual (Grieco, 2015).

### A Policy for Social Entrepreneurship

Governments have important roles in any organizational undertaking (e.g., Gartner, 1985; Austin et al., 2006; Minniti, 2008; Lerner, 2009); for example, they create both the legal and financial structures that organizations need to relate to. Today, governments, and their policies are perceived as particularly important for fostering entrepreneurship and economic growth (e.g., Minniti, 2008; Lerner, 2009). Government policies also have the power to influence and encourage entrepreneurial activities in areas that are perceived to be neglected, problematic, suffering from market failures or in need of new solutions (Audretsch et al., 2007; Minniti, 2008).

While many countries have, as aforementioned, transformed their welfare state systems into being more neoliberal (e.g., Roper and Cheney, 2005), governments have realized that social entrepreneurship have the potential to generate values that are not easily created by the market or public agencies (Mulgan, 2006). There is a conception that social entrepreneurs are more effective than the welfare state at creating social change (Nicholls, 2006). According to Dees (2007), this notion has emerged in light of the fact that traditional public services have, for example, been criticized for being “bureaucratic, ineffective, wasteful, too political, and antithetical to innovation…” (p. 25). Besides this, researchers claim that political decision-makers can be afraid of implementing new policies or developing new solutions themselves because they are often guided by a desire to be re-elected (Mair, 2010). Against this backdrop, it has become more common to provide government support for organizations whose activities are characterized by social entrepreneurship and to create systems generating incentives among other non-governmental organizations to work on solving social problems (Santos, 2012). As such, governments and policymakers can be important actors in creating conditions for social entrepreneurs (see also Lerner, 2009; Phillips et al., 2015; Ratten, 2017; Reynolds et al., 2017; Peterson and Schenker, 2018a; Baines et al., 2019).

In general, there are two main roles that political reforms and policies can have in relation to social entrepreneurship. First, reforms, and policies, such as legislation or grants, can create supportive environments or incentives for social entrepreneurs and organizations to develop socially oriented activities. In this case, political reforms can, according to Reynolds et al. (2017), be regarded as a policy for social innovation whose goals are to bring about social change. It may, for example, be about reducing the exclusion of different groups in society or initiating new funding strategies. Second, the reforms and policies can be understood as social innovations in themselves, which means that the design of policies and policymaking can be socially innovative in its process ( see also Ratten, 2017; Ratten and Ferreira, 2017; Reynolds et al., 2017; Schenker et al., 2021).

In research on so-called entrepreneurial processes, a distinction can be made between the concepts of social invention, social innovation and social entrepreneurship (e.g., Galindo and Méndez-Picazo, 2013; Ratten, 2017). These concepts are related to each other and describe various stages in an entrepreneurial process. The process begins with an idea of social and societal development (i.e., social invention). If the idea is developed and implemented in practice, a social innovation has taken place. The third and final step in the entrepreneurial process is to actually bring about social change. This step requires that social entrepreneurs work with people in their day-to-day activities to achieve social change in society (e.g., Phills et al., 2008). A consequence of this approach is that policies cannot be considered as social entrepreneurship in themselves. However, policies and politics can encourage social entrepreneurship by introducing political reforms (social innovations) designed to bring about social change. As such, these reforms can stimulate actors to create new organizations and activities to achieve various social goals (see Ratten, 2017). Next follows an in-depth presentation of the Swedish sport policy model.

## The Swedish Sport Policy Model

The Swedish sport policy model has three overarching characteristics. The first characteristic is that the voluntarily organized sport is strongly rooted in a popular movement tradition in which non-profit local sports clubs are united in national sports federations under the leadership of a joint umbrella organization, the Swedish Sports Confederation. The basis for the Swedish sports movement is the 3.1 million individual members who are active in at least one of the ~18,000 local sports clubs. The sports movement consists of both young and old, men, and women, as well as athletes, leaders, and supporters. Participation in clubs' sports activities is highest among young people. Statistics show that practically all young Swedes (ca. 90%) have been a member of at least one sport club before the age of 20 (SOU, 2008:59; see also Norberg, 2019). Analyzes over time show that participation in organized children's and youth sports has decreased since the beginning of the 2000s. However, club-based sports are still the most popular leisure activity among young people in Sweden (Norberg, 2018, 2019).

At the national level, the Swedish sports movement consists of 72 special sports federations, that is, nationwide, independent organizations in charge of various sports. The largest sports in Sweden, measured in the number of active participants, are football, athletics, golf, and gymnastics (Riksidrottsförbundet, 2020). At regional level, there are 19 district federations with the mission of both supporting the regional sports life and representing the interests of sport toward various administrative entities, such as municipalities.

The supreme decision-making body of the Swedish Sports Confederation is the General Assembly, which is held every 2nd year. Between the assembly meetings, the Swedish Sports Confederation is led by an executive committee and its office. Alongside the Swedish Sports Confederation there is the Swedish Sports Education Association and the Swedish Olympic Committee. The Olympic committee consists of the 41 Swedish special sports federations whose sports are part of the Olympic program. Its task is to organize and carry out the Swedish participation in the Olympic games, including preparations, selection of participants, and talent development (Norberg, 2018; see also Norberg, 2004). Furthermore, the roots of the Swedish sports movement as a popular movement tradition have created principles for how the activities are organized, and also created the democratic ideals and values on which this movement rests. Values such as openness and equality are central to the sports movement. Equally important is that membership in sports club should not be governed or limited by factors such as gender, socio-economic, ethnicity, and disability (Norberg, 2018).

The second characteristic of the Swedish sports policy model is the extensive public support at both national and local levels. The public support is based on welfare policy aims. Some highlighted aims are sports contribution to public health, democratic fostering of young people and social inclusion (see Wagnsson, 2009; Bergsgard and Norberg, 2010; Österlind and Wright, 2014; Ekholm, 2016; Fahlén and Stenling, 2016; Österlind, 2016). In many countries, providing public support for sport is aimed at promoting elite sports and success at an international level and justified on the assumptions that international success in sports will lead to prestige, national cohesion, tourism, and jobs. This can be seen in the UK where sport policy has flip flopped between “sport for sport's sake” and “sport for good” but it has rarely been able to achieve and sustain a balanced position (e.g., Brookes and Wiggan, 2009; Collins, 2010). However, such perspectives have never been prominent in Swedish sport policy (e.g., Bergsgard and Norberg, 2010; Norberg, 2018). As an illustrative example, the regulation that regulates the state support for sports lacks any writing on elite sports, instead one can read that the subsidies must promote public health, gender equality and democratic values (SFS, 1999:1177).

The government support for sports in Sweden amounts to more than SEK 2 billion and can roughly be divided into three parts: (a) organizational support for the Swedish Sports Confederation and the national sports federations; (b) subsidies to local sports clubs based on the extent of their activities for children and adolescents; and (c) various grants to sport federations and local clubs for time-limited and earmarked development projects, such as the “Lift for Sport” (Norberg, 2016, 2019). Besides the government support, there is also a municipal support for sport, which consists mainly of public funded sport facilities and grants to the local sports clubs. This local support for sport is estimated to be about SEK 10 billion annually (Norberg, 2018).

The third characteristic of the Swedish sports policy model is the close cooperation between the state and the Swedish Sports Confederation in matters regarding Swedish sport policy and the public support for the sports movement (e.g., Norberg, 2004). In Sweden, there is a long tradition of cooperation between the government and organized interest groups, often referred to as corporatism (see Rothstein, 1992; Micheletti, 1995; Premfors et al., 2009; Larsson et al., 2012; Lundberg, 2020). According to Lewin (1994), corporatism can be defined as “the officially sanctioned participation of organizations in decisions governing the affairs of the state or in their administration, or similar actions carried out by organizations on behalf of the state” (p. 66). In the field of sport, the close cooperation between the sports movement and the state is evidenced by the fact that the Swedish Sports Confederation has, since the 1930s, had a central role in the allocation and administration of the government support for sport. Thus, the Swedish Sports Confederation is not only a recipient of government support, but also in control of how the support is to be allocated within the sports movement. As a result, the executive committee of the Swedish Sport Confederation has developed a double identity. On the one hand, it is the supreme decision-making body of a popular movement in Sweden. On the other hand, it acts on behalf of the government in issues pertaining to sport policy (Norberg, 2004; SOU, 2008:59).

It may seem odd that Sweden developed a system, whereby the Swedish Sports Confederation is both recipient and at the same time responsible and in control of how the support is to be allocated. The explanation lies, as suggested by Norberg (2004, 2011), in the fact that the government support for sport in Sweden is not merely aimed at encouraging various social effects such as public health, democratic fostering, and social inclusion. Equally important is the motive for supporting and promoting the sports movement as a voluntary non-profit popular movement. Thus, in Sweden, the state has always deliberately chosen to limit its control and governance of sports in order to recognize and promote the Swedish Sports Confederation as an independent popular movement. As a result, the relationship between the sports movement and the Swedish government has always been vaguely formulated. While the sports movement has always emphasized its autonomy in policy documents, the government has limited its stated sports policy objectives to a minimum in order not to challenge the sports movement's independence. Even though this relationship has gradually developed since the early twentieth century, it has rarely been problematized or discussed at a policy level.

However, the low level of government control does not mean that the public support for sport has been unconditional. On the contrary, the state has always relied on the sports movement's own ability to shoulder responsibility and to develop it in a socially beneficial direction. Hence, the corporative cooperation that developed between the state and the sports movement during the twentieth century can be characterized as an “implicit contract,” based on trust, implicit expectations, and on mutual dependency rather than on control, formal agreements, and explicit objectives (Norberg, 2004, 2011; SOU, 2008:59).

## Current Changes in Swedish Sport Policy

Since the turn of the millennium, the Swedish sports policy model has undergone three significant changes, which will be presented next. Thereafter, we will analyze these changes in relation to neoliberalism, NPM, and social entrepreneurship.

### A Transformed Swedish Sport Policy Model

Firstly, the government support for sports has increased significantly, despite various financial crises, and economic recessions. The increased support can mainly be explained by the fact that the government's support for sport was linked to revenues from the state-regulated gambling market between 1990 and 2009. The decision to earmark parts of the surplus from the gambling market to sports was made by the parliament to compensate the sports movement for omitted increases in the government support during the financial crisis in the early 1990s. However, few could predict the expansive gambling policy that the government would bring about the years around the turn of the millennium and its effect on the public support for sport. In the years of 2000–2005 alone, the support increased from just over SEK 600 million (2000) to almost SEK 1,5 billion (2005). In 2018, the support amounted to SEK 2,1 billion. According to Norberg (2016), the increased support for sport from the gambling market can be characterized as a winning lottery ticket for the Swedish sports movement. With such a gaming political metaphor, Norberg (2016) argues that the increased support must be interpreted as an unexpected outcome of political decisions concerning gambling rather than a strategic sport policy initiative by the Swedish government.

Secondly, the structure of state support has changed. With new gambling money came new types of grants, some of which are to be classified as large-scale development project grants. The objectives of these grants have varied over time, but the overall ambition has been to encourage sports clubs to recruit new members, reduce dropouts, and to develop new activities. Moreover, all grants have had a time limit and been earmarked (Norberg, 2016). An illustrative example is the “Handshake”-grant (2004–2007), in which the sports movement was allocated SEK 1 billion over a 4-year period for recruiting new members, holding back fees, promoting sport activities among girls, fighting against drug use and intensifying sports clubs' cooperation with schools (Prop, 2003/2004:1, expenditure area 17, p. 123). After the “Handshake,” the “Lift for Sports” (2007–2019) was established with an overarching objective to help the sports movement recruit new members among children and prevent dropouts during adolescence (Prop, 2006/2007:1, expenditure area 17, p. 138). In addition to these grants, other earmarked political projects have been implemented. In 2015, a project called “Sports for newly arrived people” was launched with the aim to promote integration of refugees in the wake of the European refugee crisis (Government offices of Sweden, 2016; Norberg, 2019). In 2017, the government launched “United for daily movement” with the aim to promote physical activity in schools (Government offices of Sweden, 2017).

Thirdly, the relationship between the state and the sports movement has changed. The trust and implicit expectations that characterized Swedish sport policy during the twentieth century has gradually been replaced by regulations and formalized responsibilities. In 1999, the overarching aims for state support for sport became specified in a government regulation (SFS, 1999:1177). In 2010, the Swedish Research Council for Sport Science was appointed by the government to monitor the effects of the state support for sport (SFS, 2009:1226). Simultaneously, the Swedish Sports Confederation was given legal support for exercise authority in issues regarding the state support for sport (SOU, 1998:76; see also SFS, 2017:900). Consequently, the former implicit contract between the state and the sports movement has become explicit and formalized (Bergsgard and Norberg, 2010; Norberg, 2011, 2018). In the following two sections, these three changes will now be analyzed: first in relation to neoliberalism and NPM and then in relation to social entrepreneurship.

### From “Development of Sport” to “Sport for Development”: A Neoliberal Inspired Sport Policy

The changes that have taken place in Swedish sport policy since the turn of the millennium can be summarized as a shift from general public support with the aim to encourage the sports movement's existence, autonomy and voluntary endeavors to a more result-oriented sport policy focusing on the societal effects of the sports movement's activities: from a “thank you for being there” to a “thank you for what you accomplish.” The changes can also be described as a shift in perspective from “development of sport” to “sport for development” (Norberg, 2016).

There are reasons to interpret the changes in state sports policy as result of neoliberal governance ideals. The strong focus on the societal effects of public support for sports, emphasizes a perspective in which the sports movement becomes a means for the Swedish government's welfare policy efforts rather than a cultural phenomenon in its own right. The motives behind the state support for the sports movement are to a less extent to support a non-profit movement but rather a strategy to reduce government spending by benefiting the values of the sports movement's voluntary efforts (see van Kersbergen and van Waarden, 2004). As such, the sports movement is regarded as a tool and an instrument for welfare policy endeavors. Moreover, explicit sport policy goals (see SFS, 1999:1177), and strategies to measure and monitor policy effects, which the Swedish Research Council for Sport Science has been doing in Sweden since 2010 (SFS, 2009:1226), are as Hood (1991) suggests common management strategies in NPM and is closely related to the governance doctrine “management by objectives and results” (see also Ekholm, 2016; Fahlén and Stenling, 2016; Norberg, 2016). Also, since the large increase in support has been in form of project grants, it has meant that both sport federations and sports clubs have been forced to apply for these grants in competition with others in order to finance their ideas. This development, together with an increased focus on management by objective, is a sign that the support has become more inspired by NPM to both ensure that the financial support is used for its intended objectives, and to achieve economic efficiency (Hood, 1991; van Kersbergen and van Waarden, 2004; Thörn and Larsson, 2012). A rationale for exposing the grants to competition is that economic efficiency can be achieved as it is the sports clubs that are believed to have both the best ideas and the best chance of succeeding that receive the grants. After completion of the project, the recipients need to complete and submit a final report. This control function and accountability can be seen as further examples of how the introduction of NPM has influenced Swedish sport policy. For the sports movement, the changed sport policy has as indicated resulted in a significantly increased financial support, but at the price of a reduced autonomy.

However, the shift in Swedish sport policy can also be interpreted as an expression of network governance (see van Kersbergen and van Waarden, 2004). The premise for network governance is that modern globalized societies are so complex and interwoven that the boundaries between the sectors of society have become more blurred than before (Bromley and Meyer, 2017). The state can therefore no longer manage the public sector and provide welfare in accordance with traditional and hierarchical principles. Instead, the solution is to increase the cooperation with other actors in society, such as companies and non-profit organizations (van Kersbergen and van Waarden, 2004; Premfors et al., 2009; Hague and Harrop, 2010). From this view, the shift is a reaction to the fact that the Swedish government needs help, for example from the Swedish sports movement, to face major societal challenges, such as physical inactivity, social inequality, and social exclusion (see Norberg, 2019).

Regardless of whether the changes in the Swedish sports policy are characterized as a result of neoliberal ideals or as network governance, they are based on a fundamental shift of perspective. In the past, the government's financial support for sports was primarily to promote a positive development of the Swedish sports movement. Today, the aim is rather to secure the sports movement's contribution to Swedish society (e.g., Norberg, 2011; Ekholm, 2016; Fahlén and Stenling, 2016; Ekholm and Dahlstedt, 2017). An illustrative example of how Swedish sport policy has become more result-oriented and that the cooperation between state and sports movement has been more focused on facing major social challenges is the sport policy initiative made in the wake of the migrant and refugee crisis in 2015. The Swedish government responded promptly to the crisis by increasing its support for sport by allocating funds to the initiative labeled Sports for newly arrived people. In an extra amending budget bill (Prop, 2015/16:47), the Swedish government stated that “[d]ue to the current refugee situation, the government believes that additional support needs to be added to the sports movement to facilitate efforts for asylum seekers and to the work on establish and include the newly arrived into society” (p. 17). In sum, the initiative was for some years allocated SEK 64 million annually to facilitate the establishment of newly arrived people (Prop, 2015/16:47; Government offices of Sweden, 2016). The decision to allocate earmarked support for the sports movement for its work with social inclusion of newly arrived is thus a good example of the changed Swedish sport policy and the shift in perspective from “development of sport” to “sport for development.” Other examples that show this shift in perspective are: (1) the introduction of the three time-limited and earmarked grants (i.e., the Handshake, the Lift for Sports and the United for daily movement) that sports clubs have been needed to seek in competition with others; (2) more explicit sport policy goals (SFS, 1999:1177); and (3) that the government has appointed the Swedish Research Council for Sport Science to monitor the effects of the state support for sport (SFS, 2009:1226).

The sports movement has faced this development with mixed feelings (see e.g., Norberg, 2021). On the one hand, the Swedish Sports Confederation have gratefully accepted the new and increased grants that followed the changed structure of state support. On the other hand, many sports federations have argued that the autonomy of the sports movement has been curtailed as more and more subsidies have been earmarked for specific objectives. As a result, a new and more aggressive sport policy debate has arisen in which some actors within the sports movement have argued that the government should not control the public support for sports while others have argued that the Swedish Sports Confederation should cease to have an authority role in matters concerning the state support for sports (see Falck, 2021; Norberg, 2021). Both positions indicate that the relationship between the government and the sports movement is questioned and that the established “contract” will be renegotiated.

### The Swedish Sport Policy as an Expression of Social Entrepreneurship

Increased expectations of the social benefits of sport have, for natural reasons, meant increased focus on the social values that sport is assumed to generate. Since the turn of the millennium, and from a Swedish state perspective, it has become increasingly important that sport is beneficial for society, rather than strengthening the autonomy of the sports movement. The fact that the government increased its control of its support for sport, specified the motives for the support, and decided to allocate extra funds to earmarked development project grants (e.g., “Sport for newly arrived people”), support this argument. There are also indications that the Swedish government regards sport as both a partner and an important arena for achieving social change. This means that the organizations engaged in sporting activities with social objectives benefit from this development. On the basis of these aspects, the transformed Swedish sport policy has created and strengthened conditions for social entrepreneurship in sport.

Expressions of social entrepreneurship within the Swedish sport policy can in particular be discerned in the changed structure of the state support for sport. The mentioned political initiatives and development project grants (e.g., Lift for Sport and Sport for newly arrived people) can be regarded as (policies for) social innovations (see Reynolds et al., 2017; Schenker et al., 2021), especially since these explicitly aims to solve some identified and specified social problems in society in general (e.g., social inclusion of refugees or fight against drug abuse), and in sport specifically (e.g., recruiting more participants and keeping the costs down for children and youth sports) by creating important supportive systems for actors striving to bring about social change (see e.g., Phillips et al., 2015; Ratten, 2017; Reynolds et al., 2017).

The various socially political initiatives have improved the financial conditions for sport organizations to engage in social entrepreneurship. The importance of the government in promoting such activities can thus not be underestimated (e.g., Audretsch et al., 2007; Minniti, 2008; Lerner, 2009). By increasing the support for sport in the form of earmarked grants, the Swedish government can also be said to have given the sports movement a responsibility to solve various problems (see Ekholm, 2016; Österlind, 2016). The initiatives have simultaneously opened some doors for sports clubs to be more socially oriented in general and for social entrepreneurs in sports in particular. One possible analysis is that the government has had the same image of social entrepreneurs as the one that appears in research, that is, as being non-bureaucratic, inclined to take risk and willing to change their activities (see Mulgan, 2006; Nicholls, 2006; Dees, 2007; Mair, 2010). However, whether the government actually considers social entrepreneurs as more effective than public agencies and services in achieving social change is an empirical question that need to be researched. The impression though is that efficiency has been an important factor in the political decisions made to use the Swedish sports movement to realize certain social goals, for example, social inclusion through Sport for newly arrived people (see Government offices of Sweden, 2016).

By supporting the voluntary sector, of which the Swedish sports movement is a major part, the Swedish state can also more easily adapt to various societal changes and problems that arise. Hence, instead of expanding the public sector and the welfare society, it is, according to both the logic of neoliberalism (e.g., Roper and Cheney, 2005) and social entrepreneurship (e.g., Dees, 2007), less bureaucratic as well as more cost-effective to increase the state support for sport in form of earmarked project grants. This analysis is, for example, supported by Österlind (2016), who states that politicians consider sport and participation in sports as a cost-effective solution to societal problems caused by various social or economic inequalities (see also Ekholm, 2016; Ekholm and Dahlstedt, 2017).

However, the way in which the support for sport is allocated is not without problems, especially not in relation to social entrepreneurial sport organizations as these can be organized in other sectors than the voluntary sector (Austin et al., 2006). Because, as the state financial support for sport is allocated to the Swedish Sports Confederation, organizations that are not part of this joint umbrella organization are excluded from receiving this support. In addition, the allocation of the support for sport is also a result of conservative principles, which have favored more traditional sports clubs where sport is the main focus and goal rather than a means to achieve social change (see also Peterson and Schenker, 2018b). One interpretation of this is that the state, with its sport policy, wants one thing (i.e., support organizations whose goal is to achieve social change), but does another (i.e., support traditional sport organizations whose goal is to do sports). The political initiatives and development grants (e.g., the “Handshake,” the “Lift for sport” and “Sport for newly arrived people”), have had explicit social objectives and have created and provided opportunities for sports clubs within the Swedish Sports Confederation to engage in social entrepreneurship (see also Ratten, 2017; Reynolds et al., 2017; Schenker et al., 2021). But as evaluations of the “Handskake” and the “Lift for sport” have shown (e.g., Engström, 2008; Gerrevall et al., 2012; Hedenborg et al., 2012), the support for sport has in practice been allocated to the traditional sports and by doing so allowed sports clubs to expand their existing sporting activities. In other words, the support has been used by the sports clubs to do more of the same. If the state, on one hand, would like to increase its support for social entrepreneurs in sports or to other actors with similar aspirations, the way in which the financial support for sport is allocated needs to be reconsidered in order to include more organizations. In short, the present allocation system can be compared to a silo mentality insofar that sports clubs not part of the Swedish Sports Confederation cannot receive any government support (see SOU, 2016:13). Besides this, systems in which grants are, in an NPM-manner, sought in competition with others on a “grant market” tend to favor those who already have the experience of applying for grants, and who also have the time, energy, and desire to engage in various development projects. In the same way, the losers are those who for various reasons cannot seek or apply for grants. This problem is evident in all major political initiatives that have been implemented in Swedish sport since the turn of the millennium. Research demonstrates that the grants have, above all, been allocated to already strong sport contexts in socio-economically prosperous areas and not to the same extent to environments characterized by more difficult socio-economic conditions (Arnoldsson et al., 2019; Norberg, 2019). From this perspective, Swedish sport policy, with its explicit social objectives, has in practice not reached those areas where the needs are great. In the light of NPM, this should be seen as a failure. Because despite various efforts (e.g., earmarking project grants and appointing the Swedish Research Council to monitoring the state support for sport) to become more effective and efficient in order to achieve social change, these have not been very successful.

## Conclusions and Implications

In this paper, we have shown that Swedish sport policy is a divergent case in an international context. Like many other countries, Sweden has been affected by neoliberalism, thereby both reducing its public spending in relation to GDP and adopting new market-inspired governance doctrines (e.g., Peters, 2012; see also Larsson et al., 2012). However, the government support for sport has not been affected by any austerity policy. From this perspective, Swedish sport policy distinguishes itself from the current image of sport as a loser in a general trend of economic cutbacks (e.g., Giannoulakis et al., 2017; Parnell et al., 2017b, 2018; Widdop et al., 2018). Nevertheless, and as indicated in the paper, this does not mean that Swedish sport policy has avoided the influence of neoliberal governance doctrines, such as NPM. The expectations from the government on the sports movement have both increased and become more explicitly focused on the social beneficial effects of sport, such as public health, social inclusion, and democratic fostering. Additionally, and in line with NPM (see Hood, 1991; Boston et al., 1996), the governance and management of the sports movement has become more result-oriented and the control of the support has increased. To summarize, the transformation of Swedish sport policy can in contrast to other European countries be described as contradictory, especially since other countries have reduced their support for sport, either willingly through austerity policies (e.g., the UK), or have due to various reasons been forced into it (e.g., Greece) (see Parnell et al., 2017b). Consequently, the paper has contributed to and nuanced the ongoing academic discussion of how neoliberal policies in the public sector have affected sport organizations.

The second contribution of this paper is the analytical approach. By analyzing the development of the Swedish sport policy on the basis of NPM and social entrepreneurship, we have, as called for by Ratten (2017), provided theoretical insights to how sport policy can be understood (see also Ratten, 2019). When analyzing the changing Swedish sport policy using the two aforementioned concepts, several of the changes can be interpreted as an expression of a sport policy that promotes social entrepreneurship, but also social responsibility in general. A major focus of the increased results-oriented sport policy is on the social beneficial effects of sport. Furthermore, a substantial part of the increased support for sport can be attributed to large-scale, time-limited and earmarked project grants (e.g., “Handshake” and “Sport for newly arrived people”) whose objectives are, and have been, focused on various socially oriented objectives, such as social inclusion and democratic fostering. These initiatives can, additionally, be regarded as social innovations in that they can create better conditions for social entrepreneurs in sport (see also Ratten, 2017; Reynolds et al., 2017; Schenker et al., 2021).

Finally, and based on the arguments above, the paper contributes to a better understanding of the Swedish political conditions for social entrepreneurs in sport. The large-scale initiatives with their social objectives which have been initiated since the turn of the millennium have improved the conditions for sports clubs to engage in social entrepreneurship in sport. The initiatives have also provided incentives for sport federations and sports clubs within the Swedish Sports Confederation to become more socially oriented. This shift in Swedish sport policy can be understood in terms of winners and losers. Theoretically speaking, the winners are the sports clubs whose activities primarily are of social nature, or those sports clubs who would like their organization and activities to become more socially oriented. Currently, these sports clubs are able to apply for various project grants, and they are also gaining great legitimacy, from a sport policy perspective. Similarly, the losers are those sports clubs that are more interested in competitive sport and sporting results, rather than explicitly striving for achieving social change. A majority of the sports clubs in Sweden still tend to focus on doing competitive sport (see Stenling and Fahlén, 2016). In practice, there may very well be other winners and losers, not least based on the conditions surrounding the existing grant and support system. It can be particularly problematic for sports clubs that for various reasons are not part of the Swedish Sports Confederation. These are automatically excluded from the government support for sport. Furthermore, the strong focus on project grants that exist in Swedish sport policy is a problem for those sports clubs that lack the time, energy and resources to engage in various development projects in their clubs. However, it is perhaps these sports clubs that are in most need of public support?

## Data Availability Statement

The original contributions presented in the study are included in the article/supplementary material, further inquiries can be directed to the corresponding author.

## Author Contributions

All authors listed have made a substantial, direct and intellectual contribution to the work, and approved it for publication.

## Conflict of Interest

The authors declare that the research was conducted in the absence of any commercial or financial relationships that could be construed as a potential conflict of interest.

## Publisher's Note

All claims expressed in this article are solely those of the authors and do not necessarily represent those of their affiliated organizations, or those of the publisher, the editors and the reviewers. Any product that may be evaluated in this article, or claim that may be made by its manufacturer, is not guaranteed or endorsed by the publisher.
